# Effects of physical activity on depressive and anxiety symptoms of women in the menopausal transition and menopause: a comprehensive systematic review and meta-analysis of randomized controlled trials

**DOI:** 10.1186/s12966-025-01712-z

**Published:** 2025-01-24

**Authors:** Hongyu Yue, Yijiao Yang, Fangfang Xie, Jiahe Cui, Yang Li, Mengran Si, Shanshan Li, Fei Yao

**Affiliations:** 1 Shanghai Municipal Hospital of Traditional Chinese Medicine, Shanghai University of Traditional Chinese Medicine, Shanghai, China; 2 Shanghai Minhang Hospital of Intergrated Traditional Chinese and Western Medicine, Shanghai, China; 3 School of Acupuncture-Moxibustion and Tuina, Shanghai University of Traditional Chinese Medicine, Shanghai, China

**Keywords:** Physical activity, Depression, Anxiety, Menopause, Meta-analysis

## Abstract

**Background:**

Depression and anxiety may significantly affect women in the menopausal transition and menopause. In addition to traditional treatment strategies such as hormone therapy, antidepressants, and psychotherapy, physical activity (PA) have been increasingly studied, but there is no consensus about their role in menopausal women with depression and anxiety.

**Objective:**

The current study aimed to evaluate the effect of PA on the severity of depressive (DS) and anxiety (AS) symptoms in women during the menopausal transition and menopause.

**Methods:**

We searched for relevant published studies in PubMed, Embase, Web of Science, Cochrane Library, and CINAHL prior to 8 April 2024, focusing on randomized controlled trials documenting the effect of physical activity on DS and AS, and assessed study quality using the Newcastle–Ottawa Scale.

**Results:**

The data used for meta-analysis were derived from 21 studies (DS, *n* = 9; AS, *n* = 1; DS and AS combined, *n* = 11) involving 2020 participants. The results showed that PA groups demonstrated a statistically significant effect of depressive symptoms versus controls (DS [SMD: -0.66, 95% CI: -0.99 to -0.33; *P* < 0.001]; AS [SMD: -0.55, 95% CI: -0.82 to -0.27; *P* < 0.001]). As subgroup analyses demonstrated, physical exercise also reduced depressive symptom of women in menopausal status (SMD =-0.56, 95% CI: −0.96 to − 0.17, *p* = 0.006, I^2^ = 69%), postmenopausal status (SMD =-0.94, 95% CI: −1.46 to − 0.42, *p* = 0.0004, I^2^ = 94%), and both in menopausal transition and postmenopausal status (SMD =-0.30, 95% CI: −0.49 to − 0.12, *p* = 0.001, I^2^ = 0%), while it only reduced anxiety symptom of postmenopausal women (SMD =-0.96, 95% CI: −1.49 to − 0.43, *p* = 0.0004, I^2^ = 89%). Low-intensity and moderate-intensity exercise both produced increasingly benefits over depressive and anxiety symptoms. However, there is no statistically significant effect of exercise intensity on both depressive symptom and anxiety symptom.

**Conclusion:**

Physical activities with low to moderate intensity can impart remarkable improvements for managing menopausal women with depression and anxiety.

**Supplementary Information:**

The online version contains supplementary material available at 10.1186/s12966-025-01712-z.

## Introduction

Women undergo a range of physical and psychological changes during menopausal transition, encompassing vasomotor symptoms, mood disturbances, sleep problems, genitourinary problems, and other troubling illnesses that reduce the quality of life [1, 2]. Evidence suggests that women are at high risk of experiencing depression or anxiety during the menopausal transition, attributed to varying endogenous estrogen levels [3–5]. In menopausal women, the likelihood of experiencing anxiety and depression stands at 12.62% and 25.99%, potentially tripling the pre-menopausal levels [6]. Researches indicate that women undergoing menopausal transition are linked to significantly worse quality of life and increased losses in work productivity and healthcare resources [7, 8]. Consequently, there is a vigorous pursuit of scientific and effective strategies to alleviate depressive and anxiety symptoms of women in the Menopausal Transition.

Physical activity (PA) is defined as any bodily movement produced by skeletal muscles that requires expenditure of energy greater than resting levels. Physical activity results in a range of health benefits on health and well being, decreasing the risk for coronary artery disease, hypertension, diabetes mellitus, obesity, and osteoporosisa [9]. Some clinical and epidemiological studies have verified the significant impact of physical activity in treating mental health conditions, especially depression and anxiety [10–12]. Besides, studies consistently noted a significant and beneficial effect of physical activity on mental health in menopausal women [13–15]. A longitudinal observational research indicates a reduced probability of enduring significant depressive symptoms over a decade in women who exercise at moderate intensity regularly [16]. Concerning the mode of exercise, it has been demonstrated that aerobic exercise enhances depression, insomnia among menopausal wome [17]. Six months of aerobic exercise, in contrast to inactive women, aid in reducing common menopausal symptoms such as night sweats, mood fluctuations, and irritability [18].

In the last decade, there has been a noticeable rise in the quantity of published PA intervention trials among menopausal women. The small number of studies contributing to the pooled analyses and degree of heterogeneity among the included studies results in limited information and overall strength of previous review findings about effect of PA on depressive or anxiety among menopausal women. To date, there has been few systematical review of all types of PA on both depressive and anxiety outcomes among menopausal women. This study aimed to systematically review and analyze the overall findings regarding the efficacy of all types of physical activity for alleviating depressive and anxiety symptoms of women during the menopausal transition and explored the differences between varied menopause status and physical activity intensity.

## Materials and methods

### Literature information sources and search strategy

The Preferred Reporting Items for Systematic Reviews and Meta-Analyses (PRISMA) [19] was rigorously adhered to in this systematic review and meta-analysis. The study was registered in the international prospective register of systematic reviews (PROSPERO) (ID: CRD42024531437). Two researchers (HYY and FFX) independently searched and reviewed for relevant articles published up to 8 April 2024, which were all cited in five electronic database: PubMed, Embase, Web of Science, Cochrane Library and Cumulative Index of Nursing and Allied Health Literature (CINAHL). Te full search strategy is documented in the Supplementary file 1 and consisted of three modules in the search term: physical activity, menopause and mood disorder.

### Inclusion and exclusion criteria

The inclusion criteria were as follows: (1) Studies presented available original data; (2) Human research; (3) Randomized controlled trials; (4) Articles with populations in perimenopause, menopause, postmenopause, or climacteric depressive (DS) and anxiety (AS) symptoms; (5) Articles with interventions including aerobic, resistance, walking, water exercise, rotational vibration training, tai chi, circuit training, interval, or combined training with reporting parameters such as frequency, intensity, type and time.

The exclusion criteria were as follows: (1) Animal studies; (2) Articles classified as book chapters, conference abstracts, case reports, case series, letters, comments, interviews, and uncontrolled clinical trials; (3) Articles with populations experiencing menopausal symptoms due to other medical conditions, or in premenopause, or with other severe or chronic medical diseases, or psychiatric conditions requiring pharmacologic interventions; (4) Articles with interventions including undefined type of physical activity or any non-exercise interventions combined with physical activity; (5) Articles were unavailable to supply numerical data generated by specified tools or insufficient information for calculation; (6) Articles presented repetitive data.

### Data extraction

Two researchers (HYY and FFX) individually extracted data for precision and uniformity. Every study that might qualify underwent an independent assessment for the complete text, considering both inclusion and exclusion standards. To prevent the duplicate data occurred, literature that had been replicated was incorporated just a single time. When the two researchers disagreed, a conclusive agreement was achieved through team discussion and the involvement of a third researcher (JHC). Eligible randomized controlled trials were all selected from original clinical researches and other meta-analyses. Data extracted from the selected articles encompassed: title of the article, author, year, study location, patients’ characteristics (sample size, mean age, menopausal stage, BMI, medical treatment), intervention characteristics (exercise protocols, duration, intensity, intervals), outcome variables (the rating scale used to assess DS or AS, the primary endpoint value). We opted to obtain the data from the corresponding authors of studies when the methodology was unclear or when data were provided in a form unsuitable for meta-analysis. If a study reported high, moderate, and low levels of PA, data about the estimates of all levels were collected.

### Risk-of-bias assessment

Two researchers (HYY and FFX) assessed the included RCTs for risk of bias using Cochrane risk of bias assessment tool (5.1.0) [20] to assess random sequence generation (selection bias), allocation concealment (selection bias), blinding of participants and personnel (performance bias), blinding of outcome assessment (detection bias), incomplete outcome data (attrition bias), selective reporting (reporting bias) and other bias. Disagreements were resolved by consensus discussion or the third researcher (JHC). The degrees of risk of bias for each included article were assessed as “low risk” “unclear” or “high risk”. The Review Manager software (RevMan 5.3; Cochrane Collaboration, Oxford, UK) was used to perform the meta-analysis and graphic production. Results are presented in risk of bias tables for each included study.

### Data synthesis and statistical analysis

All analyses were performed using RevMan version 5.3 under the guidance of the corresponding author (FY). The primary outcome was the mean and standard deviation scores of DS and AS in every research. For each comparison of DS and AS, we calculated the standardized mean difference (SMD) and 95% confidence interval (CI). The random effect model was applied for pooling analysis, because it generates a more reliable estimate than the fixed effect analysis in cases of significant heterogeneity [21, 22]. Hedges’g method adjusted for variances caused by incorporating trials that differ in sample sizes. Heterogeneity was considered by the authors when the clinical and methodological characteristics of the studies in question were sufficiently alike for a meta-analysis to produce a significant summary. A heterogeneity test was explored using Cochran’s Q (Chi^2^ test) and *I*^*2*^ statistics. *I*^*2*^ value indicates the degree of heterogeneity among included studies as a result of variation across studies instead of sampling error. Low, moderate and high heterogeneity were defined using the I2 tests and cutoffs of 25%, 50% and 75%, respectively [23]. Testing for overall effect (Z score) was regarded as significant at *p*<0.05.

In the subgroup analysis, all data included in the meta-analysis were divided into subgroups, according to menopause status, physical activity intensity and PA types. Such analyses were used to investigate reasons of heterogeneity and to offer estimates of treatment effects for clinically relevant subgroups of patients. The results revealed the between-study heterogeneity. Forest plots were used to summarize the meta-analyses in the form of SMD, 95% confidence intervals, p-value for test of overall effect, chi-square and *I*^*2*^ test statistics. A sensitivity analysis was performed on the main results to determine whether review conclusions would have varied had the criteria been limited to studies with minimal bias risk (i.e. studies not deemed at high risk of bias in any domain and reporting acceptable methods of randomization and allocation concealment).

## Results

### Study selection and categorization

**Fig. 1:**
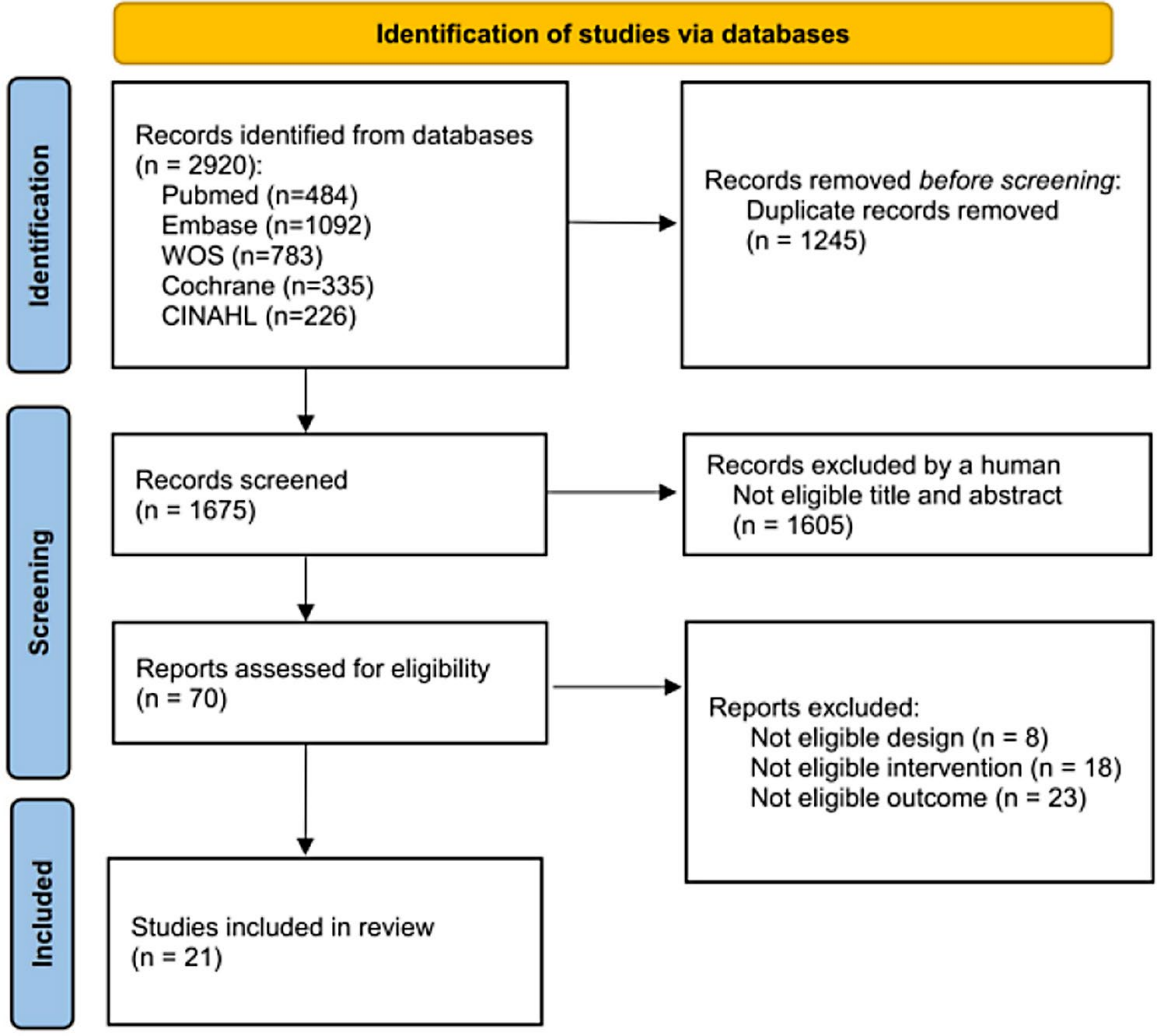
Flowchart of the selection of the studies

The systematic review resulted in 2920 records, of which 1245 duplicates were removed and 1605 articles excluded after evaluating abstract and titles. From the remaining 70 articles, we excluded 49 studies due to the following reasons: not eligible design, intervention, or outcomes. In the end, 21 studies were eligible and included in the quantitative analyses. Reasons for excluding studies at each stage of the literature screening are reported in Fig. 1.

### Summary of study characteristics

Twenty-one studies were included (DS, *n* = 9; AS, *n* = 1; DS and AS combined, *n* = 11). The included studies comprised a total of 2020 participants and 21 experimental arms (*n* = 1990 DS [20 arms]; 1411 AS [13 arms]). The general characteristics of the studies are presented in Tables 1 and 2. Studies were published between 2006 and 2022 and were conducted in Iran [24], Brazil [25], Spain [26–28], France [29], USA [17, 30–33], China [34–38], Japan [39, 40], Finland [41], Korea [42], and Turkey [43]. Sample size for each study ranged from 30 to 236, and 2020 menopausal women aged over 40 years old were recruited for all included studies, including 990 participants in the experimental group and 1030 participants in the control group. According to the modes of menopause, there were postmenopause [24–30, 32, 35, 36, 40, 43], menopause [31, 37, 38, 41, 42], Menopausal transition or postmenopause [33, 39], Late perimenopause or postmenopause [17] and perimenopause [34]. The interventions were all based on physical exercise and ranged in duration from 3 to 52 weeks, with interventions ranging from 70 to 450 min per week. The intensity of PA was based on heart rate max, VO^2^ max, heart rate reserve or estimated metabolic equivalent of task (METs) [44, 45]. Depressive symptoms were assessed with the BDI [24, 25, 29, 31, 35, 43], the BSI [30, 32], the GDS [28, 40], the HADS [26, 27], SCL-95-R [42], the PHQ [17, 33], the Kupperman Scale [37], the WHQ [41] and the SDS [34, 36, 39]. Anxiety symptoms were assessed with the BSI [30, 32], the GAD [17, 33], the BAI [25], the HAMA [38], the HADS [26, 27] the SCL-95-R [42], the WHQ [41], the SAS [36] and the Kupperman Scale [37].

**Table 1:** Baseline characteristics of the included randomized controlled trials about depressive symptoms

Author, year	Study location	Menopausal stage	Age, y	Active treatment	Duration, wk	Time/Intensity	Control treatment	Measures	Sample size active	Mean ± SD	Sample size control	Mean ± SD
Abedi et al. 2015	Iran	Postmenopausal	50–75	Walking	12w	Low intensity	No intervention	Beck Depression Inventory (BDI)	49	13.7 ± 5	48	19.6 ± 4.79
Afonso et al. 2012	Brazil	Postmenopausal	42–58	Yoga	2/w, 16w	60 min	Wait-list	Beck Depression Inventory (BDI)	15	11.0 ± 1.9	15	14.8 ± 1.9
Aibar-Almazán et al. 2017	Spain	Postmenopausal	50–75	Pilates	2/w, 12 w	60 min	No intervention	Hospital anxiety and depression scale (HADS)	55	3.98 ± 2.93	52	6.81 ± 3.6
Bernard et al. 2105	France	Postmenopausal	40–63	Walking	2/w, 6w	40 min, 40–75% maximal heart rate, moderate intensity	Wait-list	Beck Depression Inventory (BDI)	61	7.74 ± 0.77	60	10.52 ± 0.78
Bowen et al. 2006	USA	Postmenopausal	50–65	Aerobic exercise	5/w, 52w	45 min, 60–75% of VO^2^ max, moderate intensity	Stretching	Brief Symptom Inventory (BSI)	86	94.31 ± 10.4	86	93.45 ± 8.03
Carcelén-Fraile et al. 2022	Spain	Postmenopausal	60–70	Qigong	2/w, 12w	60 min	No intervention	Hospital anxiety and depression scale (HADS)	57	7.70 ± 3.23	60	10.07 ± 3.16
Elavsky et al. 2007	USA	Menopausal	40–62	Walking	3/w, 16w	60 min, 60–75% of the heart rate reserve (HRR), moderate intensity	Wait-list	Beck Depression Inventory (BDI)	63	6.38 ± 4.94	39	8.11 ± 7.64
Gao et al. 2016	China	Perimenopausal	40–62	Square dance	5/w, 12w	60–90 min	No intervention	Self-rating depressive scale (SDS)	26	0.15 ± 0.08	24	0.02 ± 0.09
Hu et al. 2017	China	Postmenopausal	45–60	Walking	3/w, 16w	60 min, 60% heart rate reserve, moderate intensity	Wait-list	Beck Depression Inventory-21 (BDI)	40	2.25 ± 1.24	40	3.85 ± 2.14
Imayama et al. 2011	USA	Postmenopausal	40–75	Aerobic exercise	5/w, 52w	45 min, 70–85% of maximal heart rate, moderate-to-vigorous intensity	Wait-list	Brief Symptom Inventory-18 (BSI-18)	117	48.1 ± 9.8	87	48.4 ± 9.6
Kai et al. 2016	Japan	Menopausal transition or postmenopausal	45–55	Stretching	7/w, 3w	10 min	Wait-list	Self-rating depressive scale (SDS)	20	35.8 ± 9.3	20	41.6 ± 7.3
Li et al. 2022	China	Postmenopausal	> 40	Baduanjin	5/w, 16w	45 min	Er Xian Decoction	Self-rating depressive scale (SDS)	17	47.06 ± 1.81	15	47.07 ± 2.19
Luoto et al. 2012	Finland	Menopausal	45–65	Aerobic training	4/w, 12w	50 min, 64-80% of maximal heart rate, moderate intensity	No intervention	Menopause-specific quality of life score (WHQ)	74	0.12 ± 0.2	77	0.22 ± 0.21
Newton et al. 2014	USA	Menopausal transition or postmenopausal	> 60	Yoga	2/w, 12w	90 min	No intervention	Patient Health Questionnaire-8 (PHQ-8)	99	-0.8 ± 3.55	133	0.1 ± 3.53
Noh et al. 2020	Korea	Menopausal		SaBang-DolGi walking	3/w, 12w	60 min	Usual care	Korea Symptom-Checklist-90-Revision (SCL-95-R)	21	43.57 ± 7.02	19	45.84 ± 8.94
Sen et al. 2020	Turkey	Postmenopausal	50–65	Whole-body vibration	3/w, 24w	20–60 min	No intervention	Beck depression inventory (BDI)	15	9 ± 3.3	18	15.9 ± 6.5
Sternfeld et al. 2014	USA	Late perimenopausal or postmenopausal	40–65	Exercise training	3/w, 12w	40–60 min, 50–70% of heart rate reserve, moderate intensity	No intervention	Patient Health Questionnaire-8 (PHQ-8)	78	-0.9 ± 3.38	135	0.1 ± 3.53
Takahashi et al. 2019	Japan	Postmenopausal	45–55	increased physical activities	8 w	>=3METs, moderate to vigorous intensity	No intervention	Geriatric Depression Scale (GDS)	19	2.9 ± 1.7	19	2.9 ± 2.6
Villaverde Gutiérrez et al. 2012	Spain	Postmenopausal		Exercise training	2–3/w, 12w	50–60 min, 50–85% maximum heart rate reserve, moderate intensity	No intervention	Geriatric Depression Scale (GDS)	27	12.11 ± 2.4	30	15.05 ± 2.6
Zhao et al. 2020	China	Menopausal	50–70	24 forms taichi	3/w, 48w	60 min, 55–65% maximum heart rate reserve, moderate intensity	No intervention	Kupperman Scale	36	0.8 ± 0.7	38	1.8 ± 0.9

**Table 2:** Baseline characteristics of the included randomized controlled trials about anxiety symptoms

Author, year	Study location	Menopausal stage	Age, y	Active treatment	Duration, wk	Intensity	Control treatment	Measures	Sample size active	Mean ± SD	Sample size control	Mean ± SD
Afonso et al. 2012	Brazil	Postmenopausal	50–75	Yoga	2/w, 16w	60 min	Wait-list	Beck Anxiety Inventory (BAI)	15	8.8 ± 1.9	15	13.5 ± 1.9
Aibar-Almazán et al. 2017	Spain	Postmenopausal	50–75	Pilates	2/w, 12 w	60 min	No intervention	Hospital anxiety and depression scale (HADS)	55	4.76 ± 3.73	52	9.37 ± 3.52
Bowen et al. 2006	USA	Postmenopausal	40–63	Aerobic exercise	5/w, 52w	45 min, 60–75% of VO^2^max, moderate intensity	Stretching	Brief Symptom Inventory (BSI)	87	94.36 ± 10.94	86	95.09 ± 8.16
Carcelén-Fraile et al. 2022	Spain	Postmenopausal	50–65	Qigong	2/w, 12w	60 min	No intervention	Hospital anxiety and depression scale (HADS)	57	5.68 ± 3.53	60	8.83 ± 4.83
Han et al. 2015	China	Menopausal	40–62	Yoga	14/w, 12w	30 min	Auricular plaster	Hamilton Anxiety Scale (HAMA)	15	14.36 ± 3.85	15	15.19 ± 3.99
Imayama et al. 2011	USA	Postmenopausal	40–62	Aerobic exercise	5/w, 52w	45 min, 70–85% of maximal heart rate, moderate-to-vigorous intensity	Wait-list	Brief Symptom Inventory-18 (BSI-18)	117	43.0 ± 6.9	87	45.3 ± 8.7
Li et al. 2022	China	Postmenopausal	40–55	Baduanjin	5/w, 16w	45 min	Er Xian Decoction	Self-anxiety scale (SAS)	17	46.65 ± 2.28	15	50.55 ± 2.06
Luoto et al. 2012	Finland	Menopausal	> 60	Aerobic training	4/w, 12w	50 min, 64-80% of maximal heart rate, moderate intensity	No intervention	Menopause-specific quality of life score (WHQ)	74	0.17 ± 0.26	77	0.19 ± 0.22
Newton et al. 2014	USA	Menopausal transition or postmenopause	50–65	Yoga	2/w, 12w	90 min	No intervention	Generalized Anxiety Disorder-7 (GAD-7)	101	-0.7 ± 3.85	135	-0.1 ± 3.26
Noh et al. 2020	Korea	Menopausal	45–55	SaBang-DolGi walking	3/w, 12w	60 min	Usual care	Korea Symptom-Checklist-90-Revision (SCL-95-R)	21	48.1 ± 6.56	19	48.47 ± 10.68
Sternfeld et al. 2014	USA	Late perimenopausal or postmenopausal		Exercise training	3/w, 12w	40–60 min, 50–70% of heart rate reserve, moderate intensity	No intervention	Generalized Anxiety Disorder-7 questionnaire (GAD-7)	82	-0.8 ± 3.47	135	-0.1 ± 3.26
Zhao et al. 2020	China	Menopausal	50–70	24 forms taichi	3/w, 48w	60 min, 55–65% maximum heart rate reserve, moderate intensity	No intervention	Kupperman Scale	36	1.8 ± 1.3	38	2.9 ± 1.7

### Quality assessment

**Fig. 2:**
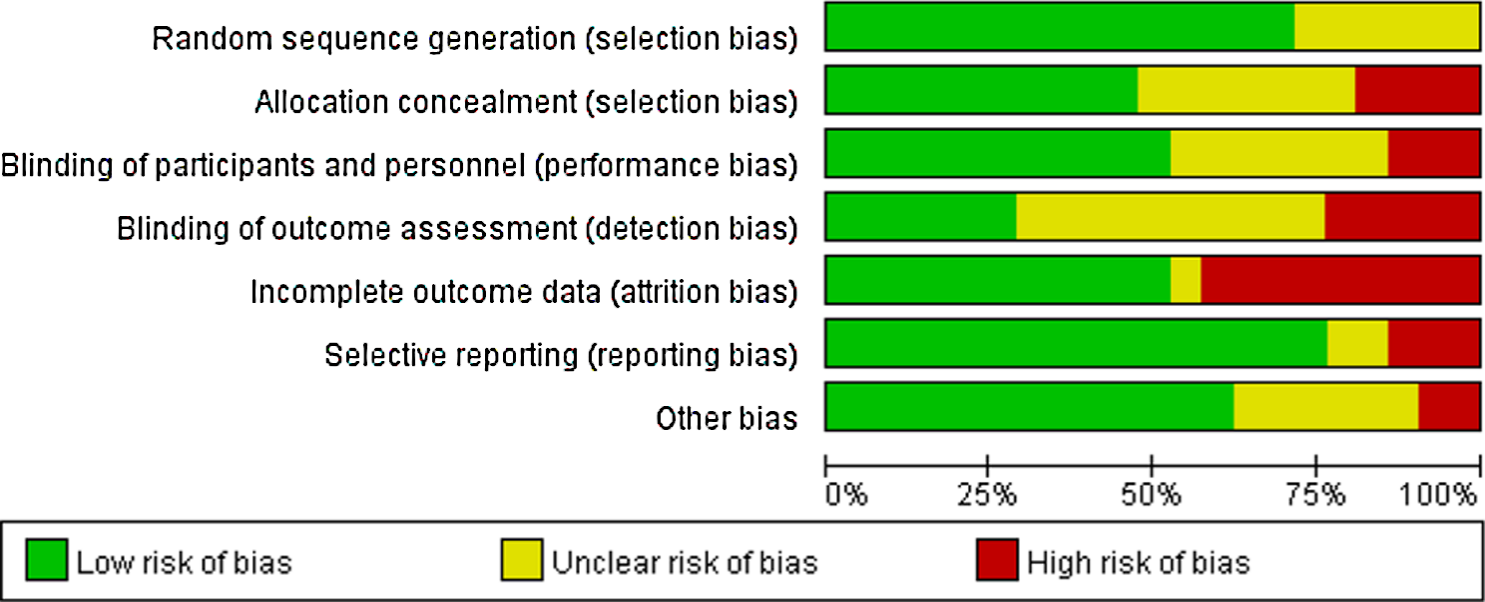
Risk of bias graph

**Fig. 3:**
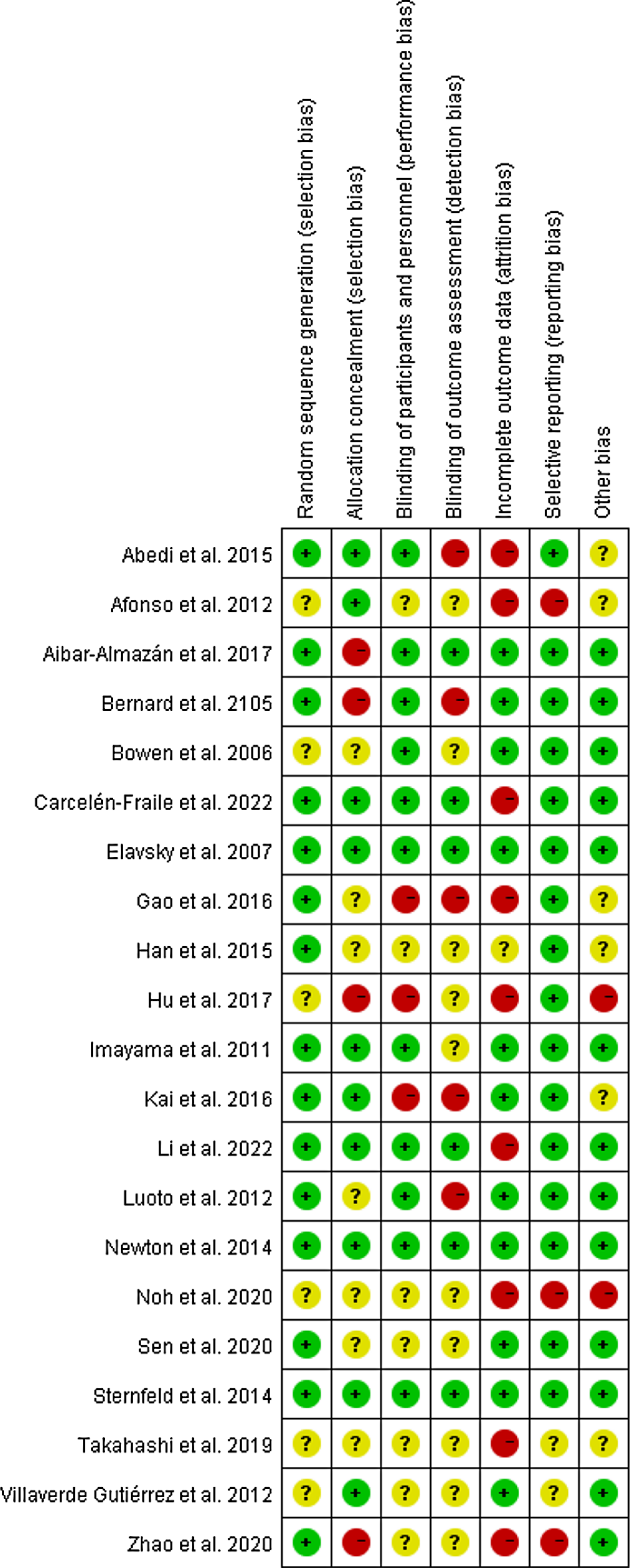
Risk of bias summary

The quality assessment showed that around 67% of the studies had some concerns or a high risk of bias (Fig. 2). Most high risk of bias found in included studies is inadequate allocation concealment [26, 29, 35, 37], inappropriate method of blinding [24, 29, 34, 35, 39, 41] and incomplete outcome data [24, 25, 27, 34–37, 40, 42] (Fig. 3). Physical activity interventions faced limitations with blinding methods and participant retention affecting attrition and adherence to protocols. Our meta-analyses revealed significant heterogeneity in pooled data, due to inconsistent DS and AS assessments and varying PA intervention.

### Summary of study outcomes

#### Depressive outcomes

**Fig. 4:**
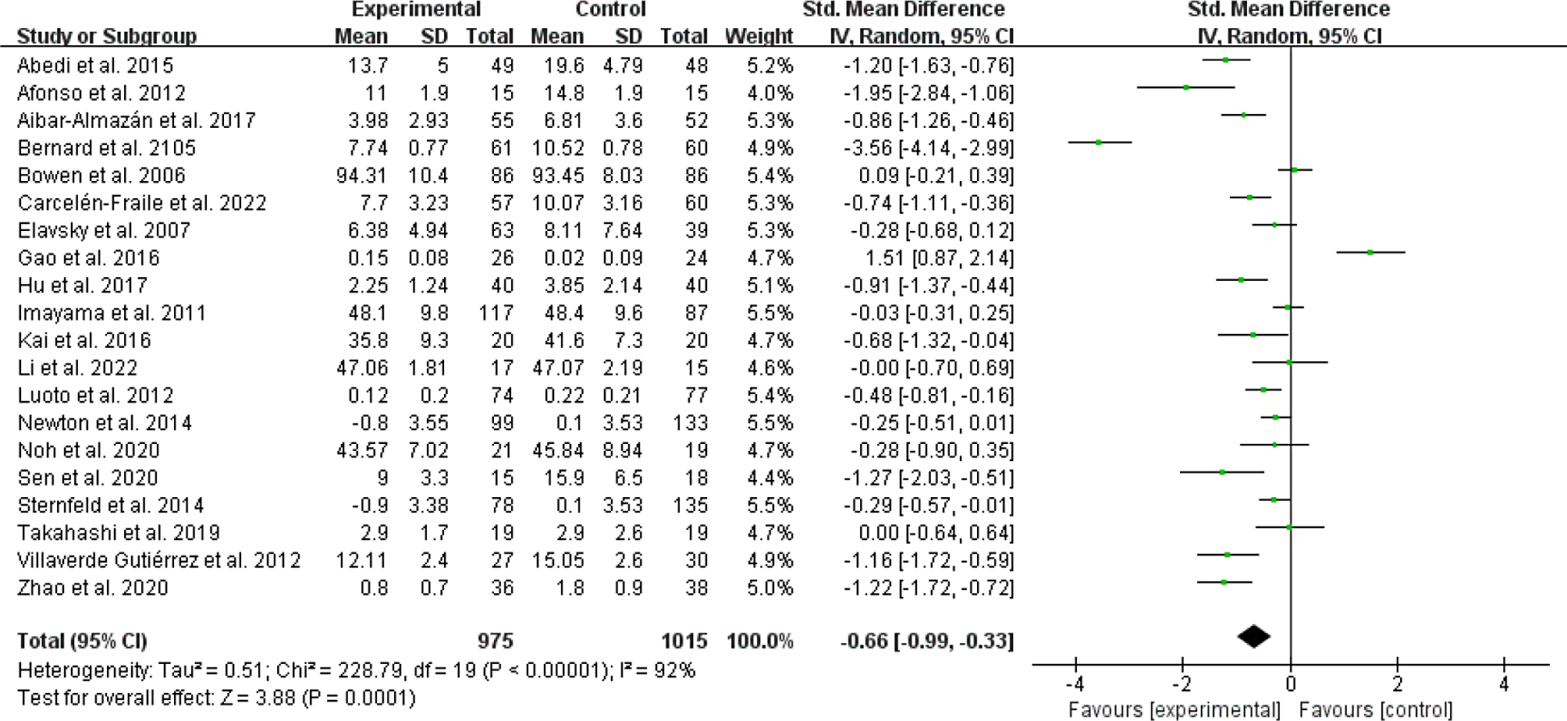
Forest plot of the depression scores

Twenty existing studies [17, 24–37, 39–43] recruited 1990 menopausal women (975 in the experimental group and 1015 in the control group) to evaluate the effects of depressive scores in menopausal women. A random-effects model was used with SMD due to different evaluation tools. The results showed that PA groups demonstrated a statistically significant effect of depressive symptoms versus controls (SMD: -0.66, 95%CI: -0.99 to -0.33; *P* < 0.001, I^2^ = 92%; *N* = 1990; Fig. 4).

#### Anxiety outcomes

**Fig. 5:**
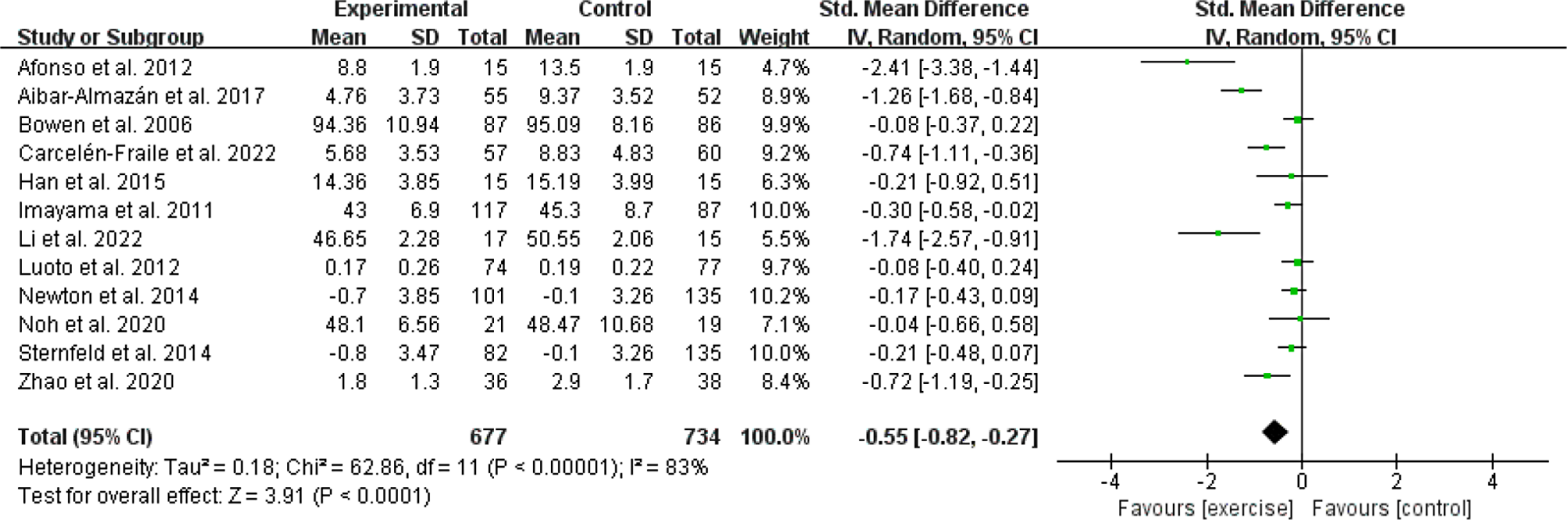
Forest plot of the anxiety scores

Twelve studies [17, 25–27, 30, 32, 33, 36–38, 41, 42] recruited 1411 menopausal women (677 in the experimental group and 734 in the control group) to evaluate the effects of anxiety scores in menopausal women. The results showed that PA groups demonstrated a statistically significant effect of anxiety symptoms versus controls (SMD: -0.55, 95% CI: -0.82 to -0.27; *P* < 0.001, I^2^ = 83%; *N* = 1411; Fig. 5).

### Subgroup analyses

#### Exercise intensity

**Fig. 6:**
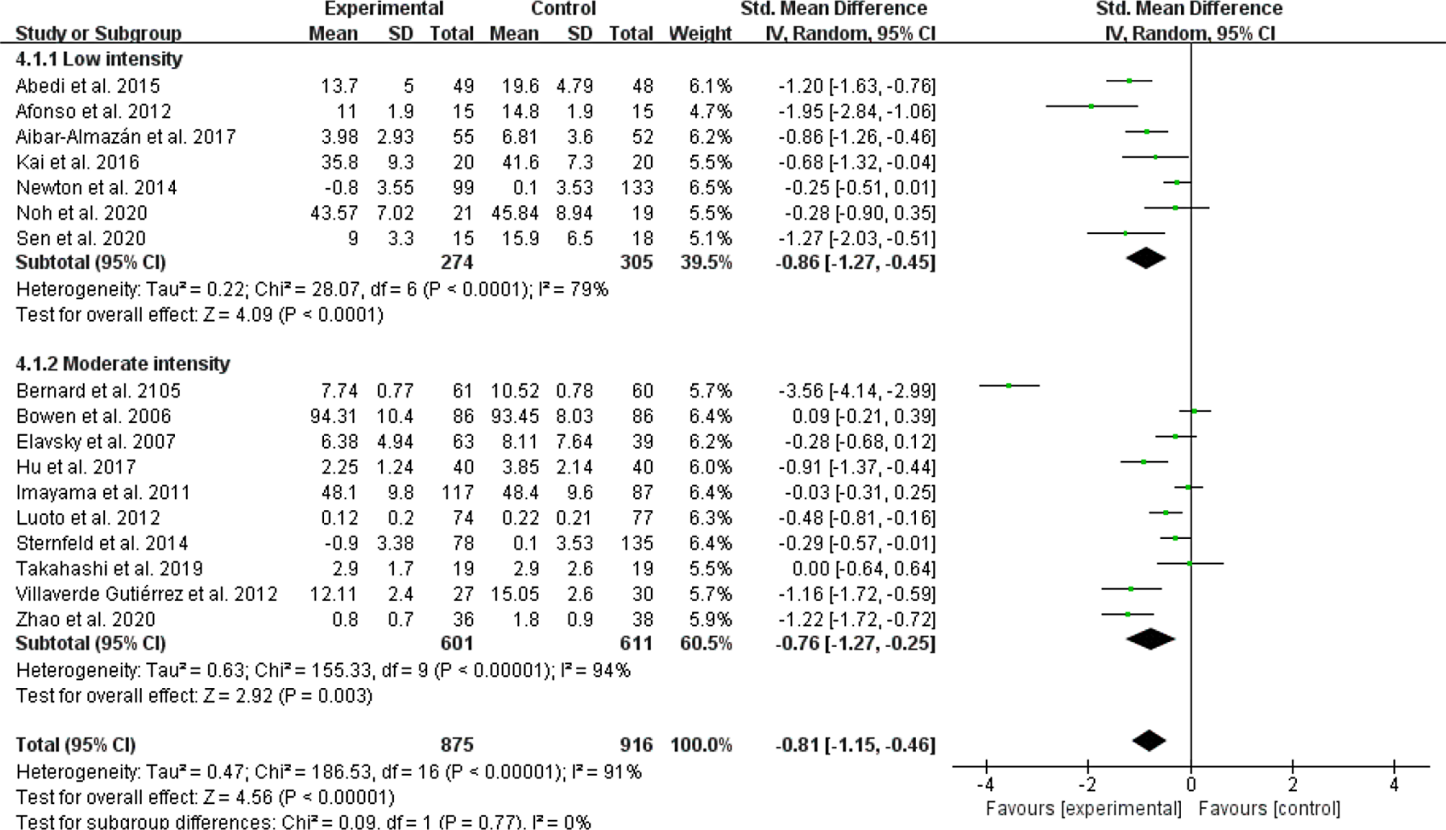
Subgroup analysis by exercise intensity evaluating depressive symptom

**Fig. 7:**
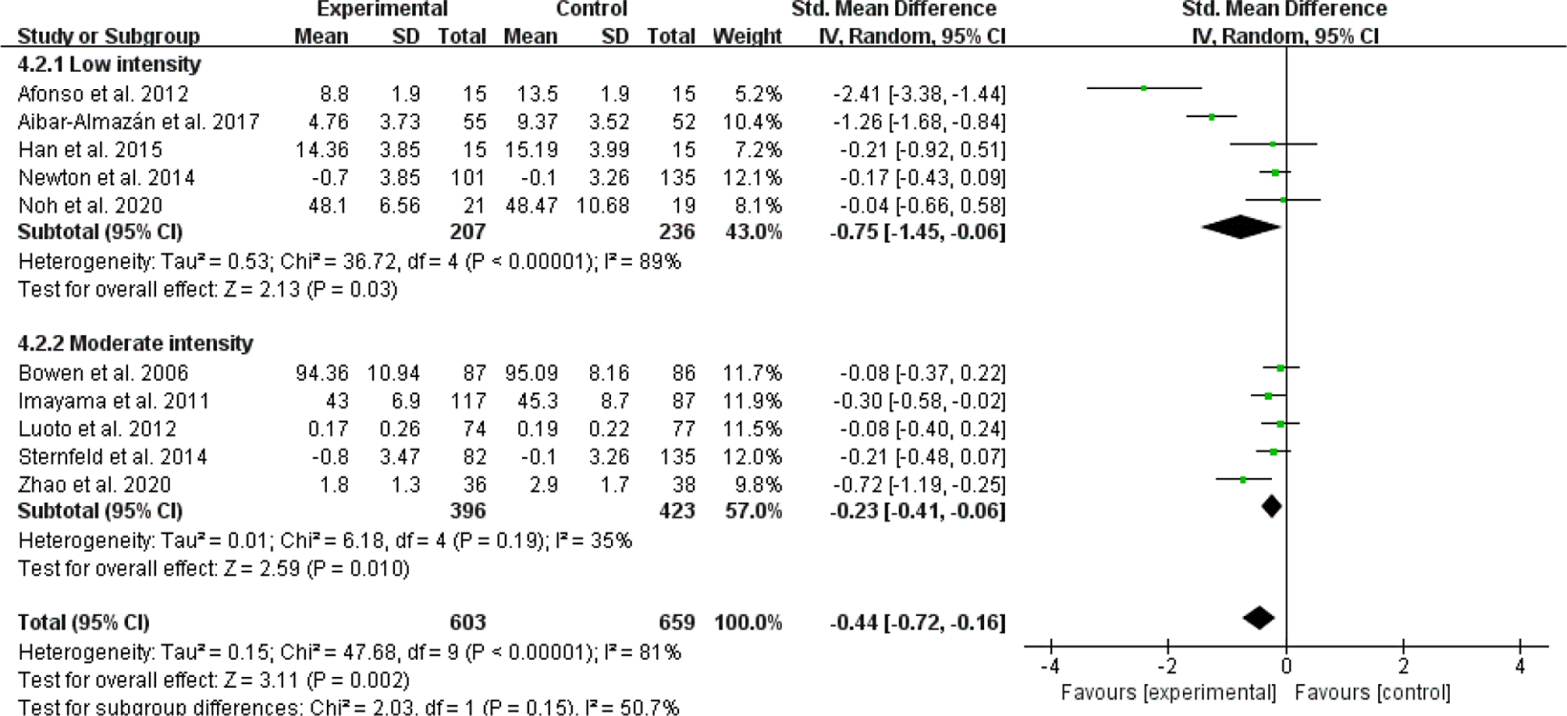
Subgroup analysis by exercise intensity evaluating anxiety symptom

Exercise intensity was classified as low intensity [24–26, 33, 38, 39, 42, 43]or moderate intensity [17, 28–32, 35, 37, 40, 41]. Moderate-intensity exercise reduced depressive and anxiety symptoms in comparison to controls (SMD =-0.76, 95% CI: −1.27 to − 0.25, *p* = 0.003, I^2^ = 94%; SMD =-0.23, 95% CI: −1.41 to − 0.06, *p* = 0.01, I^2^ = 35%; Figs. 6 and 7, respectively). Low-intensity exercise produced similar benefits over depressive and anxiety symptoms ( SMD =-0.86, 95% CI: −1.27 to − 0.45, *p* < 0.001, I^2^ = 79%; SMD =-0.75, 95% CI: −1.45 to − 0.06, *p* = 0.03, I^2^ = 89%; Figs. 6 and 7, respectively). However, there is no statistically significant effect of exercise intensity on both depressive symptom and anxiety symptom (DS: Chi^2^ = 0.09 df = 1, *P* = 0.77; AS: Chi^2^ = 2.03 df = 1, *P* = 0.15; Figs. 6 and 7, respectively).

### Menopausal state

**Fig. 8:**
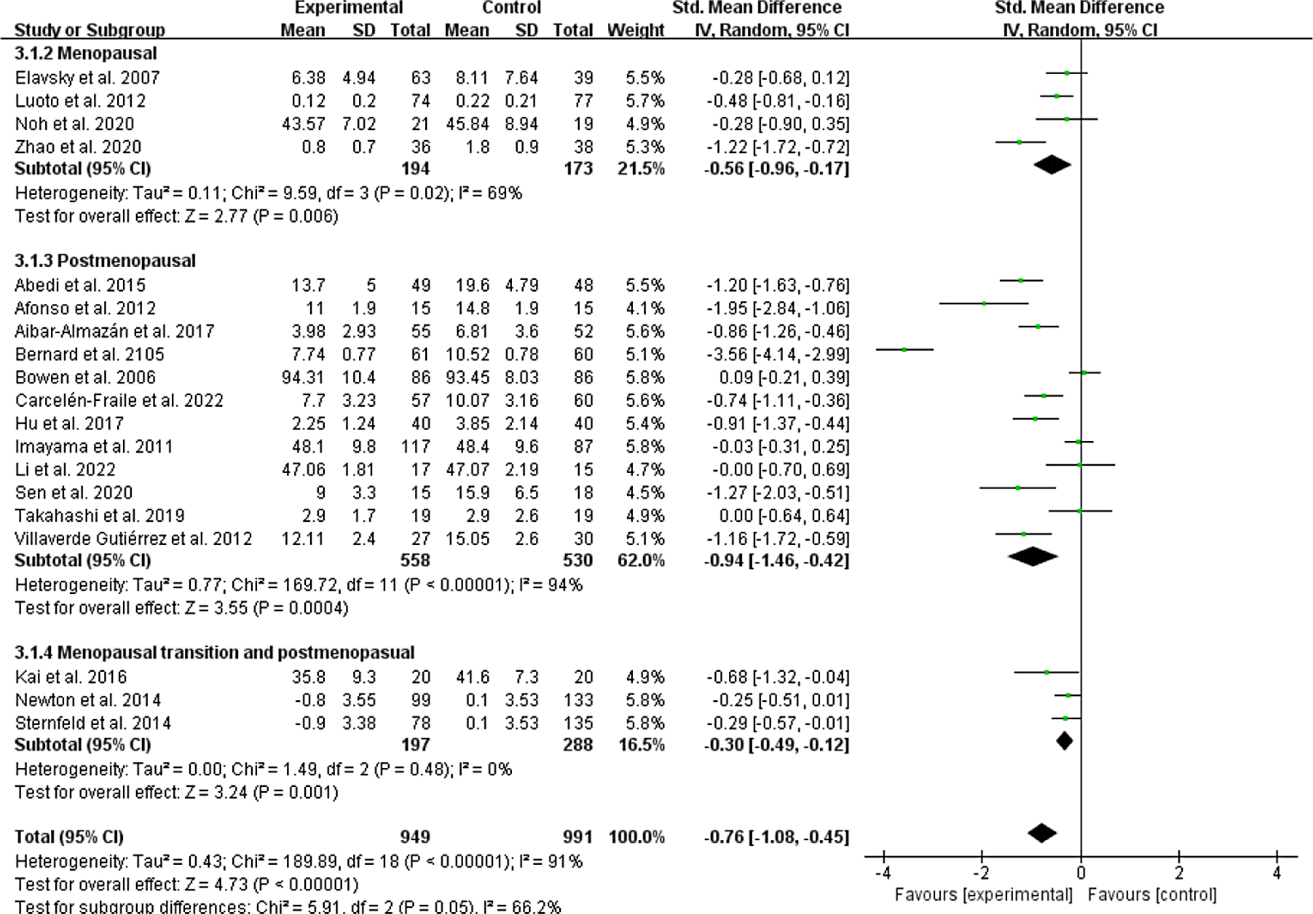
Subgroup analysis by different menopausal state evaluating depressive symptom

**Fig. 9:**
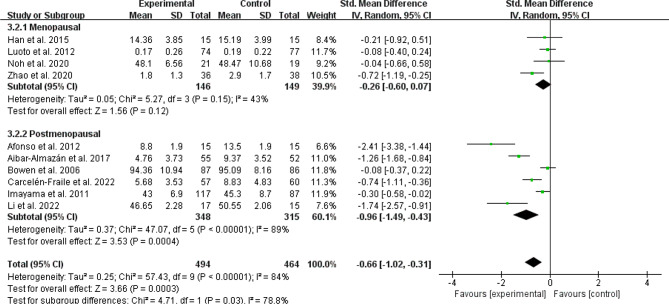
Subgroup analysis by different menopausal state evaluating anxiety symptom

Menopausal state was classified as menopausal [31, 37, 38, 41, 42], postmenopausal [24–30, 32, 35, 36, 40, 43] and both of them [17, 33, 39]. For depressive symptom, the subgroup analyses showed a statistically significant difference in all three subgroups (Menopausal: SMD =-0.56, 95% CI: −0.96 to − 0.17, *p* = 0.006, I^2^ = 69%; Postmenopausal: SMD =-0.94, 95% CI: −1.46 to − 0.42, *p* = 0.0004, I^2^ = 94%; Menopausal transition and postmenopausal: SMD =-0.30, 95% CI: −0.49 to − 0.12, *p* = 0.001, I^2^ = 0%; Fig. 8). Physical exercise also reduced anxiety symptom in postmenopausal women (SMD =-0.96, 95% CI: −1.49 to − 0.43, *p* = 0.0004, I^2^ = 89%; Fig. 9). However, no significant intervention effect on anxiety symptom was found in menopausal women (SMD =-0.26, 95% CI: −0.60 to − 0.07, *p* = 0.12, I^2^ = 43%; Fig. 9). The subgroup result showed that there was significant differences on anxiety symptom between menopausal and postmenopausal women (Chi^2^ = 4.71, df = 1, *P* = 0.03; Fig. 9).

### Sensitivity analyses

Sensitivity analysis after removing studies with included women over 65 years old found a similar result both at depression and anxiety outcomes (SMD =-0.69, 95% CI: −1.28 to − 0.10, *p* = 0.02, I^2^ = 95%; SMD =-0.38, 95% CI: −0.66 to − 0.09, *p* = 0.009, I^2^ = 71%, respectively) (Supplementary file 2, Figure S7-S8). Subsequently, a second sensitivity analysis after removing trials that included women of the control group receiving non-exercise intervention, such as medication or stretching, found that the effect of reducing depressive and anxiety symptoms still remained significant (SMD =-0.74, 95% CI: −1.10 to − 0.38, *p* < 0.001, I^2^ = 92%; SMD =-0.49, 95% CI: −0.77 to − 0.21, *p* < 0.001, I^2^ = 83%, respectively) (Supplementary file 2, Figure S9-S10). Lastly, a third sensitivity analysis evaluated the effect of the assessment tools for measuring depressive and anxiety symptoms. When only including studies using the Beck Depression Inventory (BDI) were considered, the results remained consistent (SMD =-1.51; 95% CI: −2.43, − 0.59; *p* = 0.001; I^2^ = 94%) (Supplementary file 2, Figure S11).

### Publication bias

The funnel plot served as a tool to assess the presence of publication bias in physical activity for DS and AS. The absence of some small studies in the right-hand section of the plots (outcomes with high statistical significance) for depression and anxiety scores suggested a lack of substantial evidence for small-study effects and implied that publication bias appeared not to be the source of plot asymmetry. The majority of the studies lay within the 95% confidence limits, suggesting that the results seemed be not markedly influenced by high heterogeneity between studies. (Supplementary file 2, Figure S1-S6).

## Discussion

This study is the first systematic review and meta-analysis conducted to explore the association between PA and symptoms of depression and anxiety in women during the menopausal transition and menopause. The findings suggest that moderate intensity exercises (aerobic exercise, increased PA, taichi) may lead to improvements in both depressive and anxiety symptoms in women at this stage. Additionally, various low intensity exercises such as stretching, yoga, Pilates and walking were found to lower these symptoms. However, there is a lack of research on the effectiveness of vigorous intensity exercises in reducing these symptoms. In subgroup analyses, a negative correlation between PA and depressive symptoms was observed in three groups: postmenopausal, menopausal individuals and both of them. Nevertheless, PA demonstrated a significant improvement in anxiety levels solely within the postmenopausal cohort. PA such as walking, stretching, and Chinese traditional sports were effective across all three groups. Most studies examined depressive symptoms, with only 12 studies specifically addressing anxiety symptoms. Moreover, all the decrease in anxiety is accompanied by a reduction in depression symptoms.

The results of our study are consistent with previous systematic review and meta-analyses, indicating that participation in PA may result in decreased depression and anxiety levels in adults, regardless of exercise intensity [46]. Besides, we also found low-intensity exercise may have a larger positive effect on anxiety symptom than moderate-intensity exercise. The result might be attributed to a large proportion of included articles with mind-body exercises in low-intensity subgroup. Mind-body exercise, represented by taichi, qigong, and yoga, has attracted widespread attention in the scientific literature as a way to promote physical and psychological health. A recent systematic review and meta-analysis have shown that mind-body exercises such as Pilates, yoga, tai chi, and qigong have a significant impact on reducing depression and anxiety in perimenopausal and postmenopausal women [47]. Our findings of a 12-week yoga intervention reducing anxiety symptoms align with the conclusions of prior systematic reviews and meta-analyses [48]. While there is a scarcity of meta-analyses of RCTs examining the effects of PA on reducing depression and anxiety in menopausal transition women. Our results are corroborated by previous RCTs demonstrating that PA reduces levels of anxiety and depressive symptoms [49]. This study found a correlation between low-to-moderate intensity walking and improvement in depressive symptoms among postmenopausal and menopausal women, while there is a lack of empirical research examining the association between walking and symptoms of anxiety. Noteworthy reduction in anxiety and depression was noted in women practicing Yoga. There may be a debate regarding the efficacy of aerobic exercise in improving symptoms. Traditional Chinese sports such as baduanjin, taichi, and qigong have been shown to reduce symptoms of depression and anxiety. Taken together, these findings suggest that various types of PA may alleviate symptoms of depression and anxiety in women experiencing postmenopausal and menopausal transitions.

The precise mechanism by which PA may mitigate symptoms of depression and anxiety remains unclear. Genetic predisposition and environmental factors may both contribute to this mood disorder. Our research, along with that of other scholars, demonstrates that PA can improve mood symptoms by alleviating vasomotor symptoms (VMS) and sleep disturbances [42, 50, 51]. In additional, it is posited that the mood symptoms experienced by women during menopausal stages may be influenced by various disrupting factors such as ovarian failure [52], estrogen withdrawal [53], increased levels of follicle-stimulating hormone [54], heightened neuroticism [55], and alterations in hormonal levels impacting serotonin and GABA signaling [40, 56]. At present, there is a dearth of research on the impact of these factors on the relationship between PA and mood symptoms, necessitating further investigation in subsequent studies.

This study has strengths. Our study employs a rigorous search strategy, categorize the intensity levels of PA, the inclusion of a substantial number of studies, and the use of conservative statistical methods to analyze the results. One of the primary strengths of our systematic review and meta-analysis was its comprehensive investigation of many types of PA on depressive and anxiety symptoms during peri- and post-menopausal periods. Additional strengths of the study included the incorporation of a substantial quantity of RCTs, contributing to the reliability and validity of the findings. This research also has several limitations that should be acknowledged. First, more than half of the studies exhibited a high risk of bias, attributed to the lack of precision in blinding methods and incomplete outcome data. Second, the studies included in the analysis exhibited limitations in terms of their quality. Variation in study quality contributed to the heterogeneity of findings noted in several of the meta-analyses presented in our study. The absence of standardized definitions for menopausal stage, PA assessment tools, scoring criteria for depressive and anxiety symptoms has led to significant heterogeneity in research findings. Other potential sources of heterogeneity may include the type of PA, duration of the intervention, frequency of sessions, ethnicity, level of education, employment status, lifestyle factors, body mass index, economic status, and marital status. Third, the sample size in some available studies was limited, hence, it is advisable to interpret our findings with caution. Fourth, the predominant focus of research outcomes lies in the realm of co-occurring symptoms, specifically VMS and mood disorders. other scholarly investigations predominantly center on mild-to-moderate mood disorders, with a scarcity of reviews addressing severe mood disorders. Finally, most studies do not incorporate a long-term follow-up process post-intervention.

This review may have multiple implications. First, the results of the study support the notion that various forms of exercise therapy may lead to improvement in depressive and anxiety symptoms during the menopausal transition. Second, the results highlight significant deficiencies in current research and understanding, particularly in regards to potentially advantageous treatments and evaluated outcomes. Specifically, while a majority of existing studies concentrate on low and moderate intensity PA, there is a limited number of studies examining vigorous intensity PA. Furthermore, there is a lack of sufficient research on the effects of severe mood disorders. Additional, there is a lack of uniformity in the methods used to assess depression and anxiety. Third, this review highlights the dearth of data regarding the effects of long-term follow-up in relation to menopausal symptoms. Given the chronic and recurring nature of these symptoms, it is crucial to examine the potential impact of short-term exercise interventions in altering patients’ sedentary behaviors and establishing enduring health benefits. Further studies should focus on quantifying PA intensity with objective data and uniforming the methods to assess depression and anxiety. Patients should undergo testing for sex hormones to determine the specific stages of menopause. Moreover, it is imperative to investigate the impact of varying intensities and durations of identical physical activities on the different degree of mood disorders. Additionally, we must research how different physical activities impact the same groups of people.

## Conclusion

Physical activities with low to moderate intensity might impart remarkable improvements for managing menopausal women with DS and/or AS. While menopausal status may be integral to potential clinical gains, the relationship between these variables and treatment responses remains unclear. Considerable heterogeneity among studies underscores the importance of any relative reasons of exploring other metabolic or sociodemographic factors contributing to the differences. The progression of this research and its potential application in our patient care could immensely profit from more extensive and meticulously designed studies.

## Electronic supplementary material

Below is the link to the electronic supplementary material.

Supplementary Material 1

Supplementary Material 2

Supplementary Material 3

## Data Availability

The data underlying this article will be shared on reasonable request to the corresponding authors.

## Abbreviations

AS: Anxiety Symptom
CI: Confidence Interval
CINAHL: Cumulative Index of Nursing and Allied Health Literature
DS: Depressive Symptom
MET: Metabolic Equivalent of Task
PA: Physical Activity
PRISMA: Preferred Reporting Items for Systematic Reviews and Meta-Analyses
RCT: Randomized Controlled Trial
SMD: Standardized Mean Difference
VMS: Vasomotor Symptoms
