# Supplementary material for: Effects of physical activity on depressive and anxiety symptoms of women in the menopausal transition and menopause: a comprehensive systematic review and meta-analysis of randomized controlled trials
Source: Int J Behav Nutr Phys Act. 2025 Jan 24;22:13. doi: 10.1186/s12966-025-01712-z (PMC11762881; doi:10.1186/s12966-025-01712-z)
Supplement: Supplementary file 2 — Supplementary Material 2 [file 12966_2025_1712_MOESM2_ESM.docx]

**Supplementary file 1**

**Searching strategy**

Studies were retrieved using medical subject headings combined with entry terms. The strategy combined keywords extracted from Medical Subject Headings (MeSH) or EMTREE with “OR” within the word group and “AND” to combine terms related to physical activity and menopause. We used the following terms for physical activity: Exercise; Exercises; Physical Activity; Activities, Physical; Activity, Physical; Physical Activities; Exercise, Physical; Exercises, Physical; Physical Exercise; Physical Exercises; Acute Exercise; Acute Exercises; Exercise, Acute; Exercises, Acute; Exercise, Isometric; Exercises, Isometric; Isometric Exercises; Isometric Exercise; Exercise, Aerobic; Aerobic Exercise; Aerobic Exercises; Exercises, Aerobic; Exercise Training; Exercise Trainings; Training, Exercise; Trainings, Exercise. The following terms were used for menopause: Menopause; Menopausal; Perimenopause; Perimenopausal; Postmenopause; Postmenopausal; Climacteric; Climacterics; Hot Flashes; Hot Flash; Night Sweat; Vasomotor Symptom. And subsequently we used the following terms for depressive and anxiety symptoms: Mood Disorders; Mood Disorder; Disorder, Mood; Disorders, Mood; Affective Disorders; Affective Disorder; Disorder, Affective; Disorders, Affective; Negative Emotion; Negative Mood; Depression; Depressive; Depressive Symptoms; Depressive Symptom; Symptom, Depressive; Emotional Depression; Depression, Emotional; Anxiety; Angst; Anxious; Hypervigilance; Nervousness; Anxiousness; Anguish.

**Pubmed**

1#

((((((((((((((((((((((((((Exercise[MeSH Terms]) OR (Exercise[Title/Abstract])) OR (Exercises[Title/Abstract])) OR (Physical Activity[Title/Abstract])) OR (Physical Activities[Title/Abstract])) OR (Activities, Physical[Title/Abstract])) OR (Activity, Physical[Title/Abstract])) OR (Exercise, Physical[Title/Abstract])) OR (Exercises, Physical[Title/Abstract])) OR (Physical Exercise[Title/Abstract])) OR (Physical Exercises[Title/Abstract])) OR (Acute Exercise[Title/Abstract])) OR (Acute Exercises[Title/Abstract])) OR (Exercise, Acute[Title/Abstract])) OR (Exercises, Acute[Title/Abstract])) OR (Exercise, Isometric[Title/Abstract])) OR (Exercises, Isometric[Title/Abstract])) OR (Isometric Exercises[Title/Abstract])) OR (Isometric Exercise[Title/Abstract])) OR (Exercise, Aerobic[Title/Abstract])) OR (Exercises, Aerobic[Title/Abstract])) OR (Aerobic Exercise[Title/Abstract])) OR (Aerobic Exercises[Title/Abstract])) OR (Exercise Training[Title/Abstract])) OR (Exercise Trainings[Title/Abstract])) OR (Training, Exercise[Title/Abstract])) OR (Trainings, Exercise[Title/Abstract])

2#

((((((((((((menopause[MeSH Terms]) OR (menopause[Title/Abstract])) OR (menopausal[Title/Abstract])) OR (Perimenopause[Title/Abstract])) OR (Perimenopausal[Title/Abstract])) OR (Postmenopause[Title/Abstract])) OR (Postmenopausal[Title/Abstract])) OR (Climacteric[Title/Abstract])) OR (Climacterics[Title/Abstract])) OR (Hot Flashes[Title/Abstract])) OR (Hot Flash[Title/Abstract])) OR (Night Sweat[Title/Abstract])) OR (Vasomotor Symptom[Title/Abstract])

3#

((((((((((((((((((((((((Mood Disorders[MeSH Terms]) OR (Mood Disorders[Title/Abstract])) OR (Mood Disorder[Title/Abstract])) OR (Disorder, Mood[Title/Abstract])) OR (Disorders, Mood[Title/Abstract])) OR (Affective Disorders[Title/Abstract])) OR (Affective Disorder[Title/Abstract])) OR (Disorder, Affective[Title/Abstract])) OR (Disorders, Affective[Title/Abstract])) OR (Negative Emotion[Title/Abstract])) OR (Negative Mood[Title/Abstract])) OR (Depression[Title/Abstract])) OR (Depressive[Title/Abstract])) OR (Depressive Symptoms[Title/Abstract])) OR (Depressive Symptom[Title/Abstract])) OR (Symptom, Depressive[Title/Abstract])) OR (Emotional Depression[Title/Abstract])) OR (Depression, Emotional[Title/Abstract])) OR (Anxiety[Title/Abstract])) OR (Angst[Title/Abstract])) OR (Anxious[Title/Abstract])) OR (Hypervigilance[Title/Abstract])) OR (Nervousness[Title/Abstract])) OR (Anxiousness[Title/Abstract])) OR (Anguish[Title/Abstract])

#4: #1AND #2 AND #3

**Cochrane Library**

#1 MeSH descriptor: [Exercise] explode all trees

#2 (Exercise OR Exercises OR Physical Activity OR Activities, Physical OR Activity, Physical OR Physical Activities OR Exercise, Physical OR Exercises, Physical OR Physical Exercise OR Physical Exercises OR Acute Exercise OR Acute Exercises OR Exercise, Acute OR Exercises, Acute OR Exercise, Isometric OR Exercises, Isometric OR Isometric Exercises OR Isometric Exercise OR Exercise, Aerobic OR Aerobic Exercise OR Aerobic Exercises OR Exercises, Aerobic OR Exercise Training OR Exercise Trainings OR Training, Exercise OR Trainings, Exercise):ti,ab,kw

#3: #1 OR #2

#4 MeSH descriptor: [Menopause] explode all trees

#5 (Menopause OR Menopausal OR Perimenopause OR Perimenopausal OR Postmenopause OR Postmenopausal OR Climacteric OR Climacterics OR Hot Flashes OR Hot Flash OR Night Sweat OR Vasomotor Symptom):ti,ab,kw

#6: #4 OR #5

#7 MeSH descriptor: [Mood Disorders] explode all trees

#8 (Mood Disorders OR Mood Disorder OR Disorder, Mood OR Disorders, Mood OR Affective Disorders OR Affective Disorder OR Disorder, Affective OR Disorders, Affective OR Negative Emotion OR Negative Mood OR Depression OR Depressive OR Depressive Symptoms OR Depressive Symptom OR Symptom, Depressive OR Emotional Depression OR Depression, Emotional OR Anxiety OR Angst OR Anxious OR Hypervigilance OR Nervousness OR Anxiousness OR Anguish

#9: #7 OR #8

#10: #3 AND #6 AND#9

**Web of Science**

**#1 (((((((((((((((((((((((((TS=( Exercise)) OR TS=( Exercises)) OR TS=(Physical Activity)) OR TS=(Physical Activities)) OR TS=(Activities, Physical)) OR TS=(Activity, Physical)) OR TS=(Exercise, Physical)) OR TS=(Exercises, Physical)) OR TS=(Physical Exercise)) OR TS=(Physical Exercises)) OR TS=(Acute Exercise)) OR TS=(Acute Exercises)) OR TS=(Exercise, Acute)) OR TS=(Exercises, Acute)) OR TS=(Exercise, Isometric)) OR TS=(Exercises, Isometric)) OR TS=(Isometric Exercises)) OR TS=(Isometric Exercise)) OR TS=(Exercise, Aerobic)) OR TS=(Exercises, Aerobic)) OR TS=(Aerobic Exercise)) OR TS=(Aerobic Exercises)) OR TS=(Exercise Training)) OR TS=(Exercise Trainings)) OR TS=(Training, Exercise)) OR TS=(Trainings, Exercise)**

**#2 (((((((((((TS=(Menopause)) OR TS=(Menopausal)) OR TS=(Perimenopause)) OR TS=(Perimenopausal)) OR TS=(Postmenopause)) OR TS=(Postmenopausal)) OR TS=(Climacteric)) OR TS=(Climacterics)) OR TS=(Hot Flashes)) OR TS=(Hot Flash)) OR TS=(Night Sweat)) OR TS=(Vasomotor Symptom)**

**#3 (((((((((((((((((((((((TS=(Mood Disorders)) OR TS=(Mood Disorder)) OR TS=(Disorder, Mood)) OR TS=(Disorders, Mood)) OR TS=(Affective Disorders)) OR TS=(Affective Disorder)) OR TS=(Disorder, Affective)) OR TS=(Disorders, Affective)) OR TS=(Negative Emotion)) OR TS=(Negative Mood)) OR TS=(Depression)) OR TS=(Depressive)) OR TS=(Depressive Symptoms)) OR TS=(Depressive Symptom)) OR TS=(Symptom, Depressive)) OR TS=(Emotional Depression)) OR TS=(Depression, Emotional)) OR TS=(Anxiety)) OR TS=(Angst)) OR TS=(Anxious)) OR TS=(Hypervigilance)) OR TS=(Nervousness)) OR TS=(Anxiousness)) OR TS=(Anguish)**

**#4: #1AND #2 AND #3**

**Embase**

#1 'exercise'/exp OR 'exercise'

#2 'Exercise':ab,kw,ti OR 'Exercises':ab,kw,ti OR 'Physical Activity':ab,kw,ti OR 'Activities, Physical':ab,kw,ti OR 'Activity, Physical':ab,kw,ti OR 'Physical Activities':ab,kw,ti OR 'Exercise, Physical':ab,kw,ti OR 'Exercises, Physical':ab,kw,ti OR 'Physical Exercise':ab,kw,ti OR 'Physical Exercises':ab,kw,ti OR 'Acute Exercise':ab,kw,ti OR 'Acute Exercises':ab,kw,ti OR 'Exercise, Acute':ab,kw,ti OR 'Exercises, Acute':ab,kw,ti OR 'Exercise, Isometric':ab,kw,ti OR 'Exercises, Isometric':ab,kw,ti OR 'Isometric Exercises':ab,kw,ti OR 'Isometric Exercise':ab,kw,ti OR 'Exercise, Aerobic':ab,kw,ti OR 'Aerobic Exercise':ab,kw,ti OR 'Aerobic Exercises':ab,kw,ti OR 'Exercises, Aerobic':ab,kw,ti OR 'Exercise Training':ab,kw,ti OR 'Exercise Trainings':ab,kw,ti OR 'Training, Exercise':ab,kw,ti OR 'Trainings, Exercise':ab,kw,ti

#3: #1 OR #2

#4 'menopause'/exp OR 'menopause'

#5 'Menopause':ab,kw,ti OR 'Menopausal':ab,kw,ti OR 'Perimenopause':ab,kw,ti OR 'Perimenopausal':ab,kw,ti OR 'Postmenopause':ab,kw,ti OR 'Postmenopausal':ab,kw,ti OR 'Climacteric':ab,kw,ti OR 'Climacterics':ab,kw,ti OR 'Hot Flashes':ab,kw,ti OR 'Hot Flash':ab,kw,ti OR 'Night Sweat':ab,kw,ti OR 'Vasomotor Symptom':ab,kw,ti

#6: #4 OR #5

#7 'Mood Disorder'/exp OR 'Mood Disorder'

#8 'Mood Disorders':ab,kw,ti OR 'Mood Disorder':ab,kw,ti OR 'Disorder, Mood':ab,kw,ti OR 'Disorders, Mood':ab,kw,ti OR 'Affective Disorders':ab,kw,ti OR 'Affective Disorder':ab,kw,ti OR 'Disorder, Affective':ab,kw,ti OR 'Disorders, Affective':ab,kw,ti OR 'Negative Emotion':ab,kw,ti OR 'Negative Mood':ab,kw,ti OR 'Depression':ab,kw,ti OR 'Depressive':ab,kw,ti OR 'Depressive Symptoms':ab,kw,ti OR 'Depressive Symptom':ab,kw,ti OR 'Symptom, Depressive':ab,kw,ti OR 'Emotional Depression':ab,kw,ti OR 'Depression, Emotional':ab,kw,ti OR 'Anxiety':ab,kw,ti OR 'Angst':ab,kw,ti OR 'Anxious':ab,kw,ti OR 'Hypervigilance':ab,kw,ti OR 'Nervousness':ab,kw,ti OR 'Anxiousness':ab,kw,ti OR 'Anguish':ab,kw,ti

#9: #7 OR #8

#10: #3 AND #6 AND #9

**CINAHL(Cumulative Index of Nursing and Allied Health Literature)**

S1

SU Exercise OR AB（Exercise OR Exercises OR Physical Activity OR Activities, Physical OR Activity, Physical OR Physical Activities OR Exercise, Physical OR Exercises,Physical OR Physical Exercise OR Physical Exercises OR Acute Exercise OR Acute Exercises OR Exercise, Acute OR Exercises, Acute OR Exercise, Isometric OR Exercises, Isometric OR Isometric Exercises OR Isometric Exercise OR Exercise Aerobic OR Aerobic Exercise OR Aerobic Exercises OR Exercises, Aerobic OR Exercise Training OR OR Exercise Trainings OR Training, Exercise OR Trainings, Exercise)

S2

SU Menopause OR AB (Menopause OR Menopausal OR Perimenopause OR Perimenopausal OR Postmenopause OR Postmenopausal OR Climacteric OR Climacterics OR Hot Flashes OR Hot Flash OR Night Sweat OR Vasomotor Symptom)

S3

SU Mood Disorder OR AB (Mood Disorders OR Mood Disorder OR Disorder, Mood OR Disorders,Mood OR Affective Disorders OR Affective Disorder OR Disorder Affective OR Disorders, Affective OR Negative Emotion OR Negative Mood OR Depression OR Depressive OR Depressive Symptoms OR Depressive Symptom OR Symptom, Depressive OR Emotional Depression OR Depression, Emotional OR Anxiety OR Angst OR Anxious OR Hypervigilance OR Nervousness OR Anxiousness OR Anguish)

S4: S1 AND S2 AND S3
