# Supplementary material for: Effects of physical activity on depressive and anxiety symptoms of women in the menopausal transition and menopause: a comprehensive systematic review and meta-analysis of randomized controlled trials
Source: Int J Behav Nutr Phys Act. 2025 Jan 24;22:13. doi: 10.1186/s12966-025-01712-z (PMC11762881; doi:10.1186/s12966-025-01712-z)
Supplement: Supplementary file 3 — Supplementary Material 3 [file 12966_2025_1712_MOESM3_ESM.docx]

**Supplementary file 2**

Supplementary Table S1. Funnel plot for depression scores.

Supplementary Table S2. Funnel plot for anxiety scores.

Supplementary Table S3. Funnel plot for subgroup analysis by by different menopausal state evaluating depressive symptom.

Supplementary Table S4. Funnel plot for subgroup analysis by different menopausal state evaluating anxiety symptom.

Supplementary Table S5. Funnel plot for subgroup analysis by exercise intensity evaluating depression symptom.

Supplementary Table S6. Funnel plot for subgroup analysis by exercise intensity evaluating anxiety symptom.

Supplementary Table S7. Forest plot for sensitivity analyses evaluating depression symptom after removing studies with included women over 65 years old

Supplementary Table S8. Forest plot for sensitivity analyses evaluating anxiety symptom after removing studies with included women over 65 years old

Supplementary Table S9. Forest plot for sensitivity analyses evaluating depression symptom after removing trials that included women of the control group receiving non-exercise intervention

Supplementary Table S10. Forest plot for sensitivity analyses evaluating anxiety symptom after removing trials that included women of the control group receiving non-exercise intervention

Supplementary Table S11. Forest plot for sensitivity analyses evaluating depression symptom after including trials only using the BDI.

**
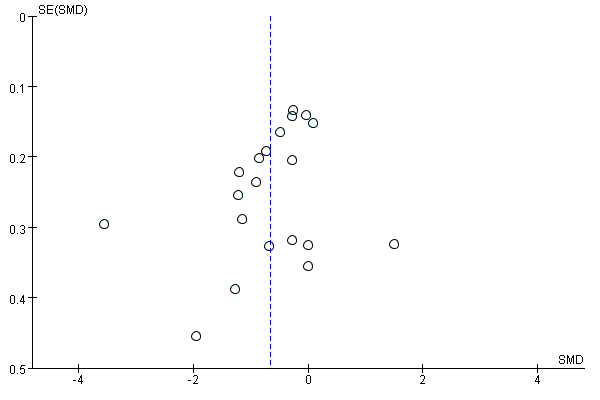
**

**Figure S1. Funnel plot for depression scores.**

**
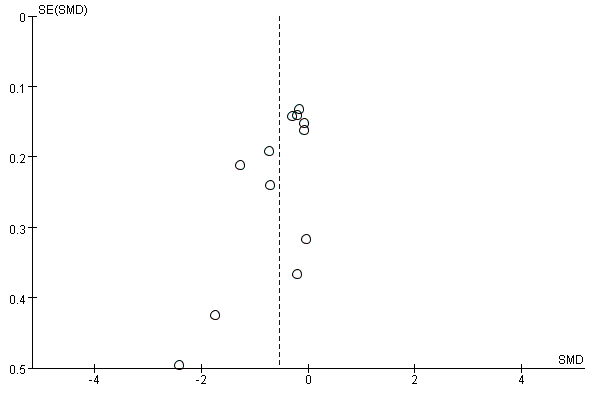
**

**Figure S2. Funnel plot for** **anxiety scores.**


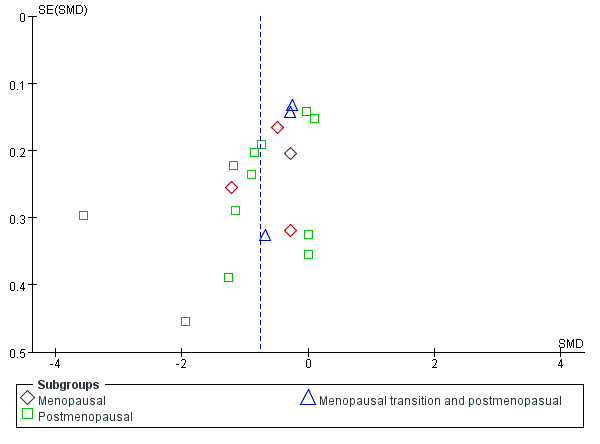


**Supplementary Table S3. Funnel plot for subgroup analysis by by different menopausal state evaluating depressive symptom.**

**
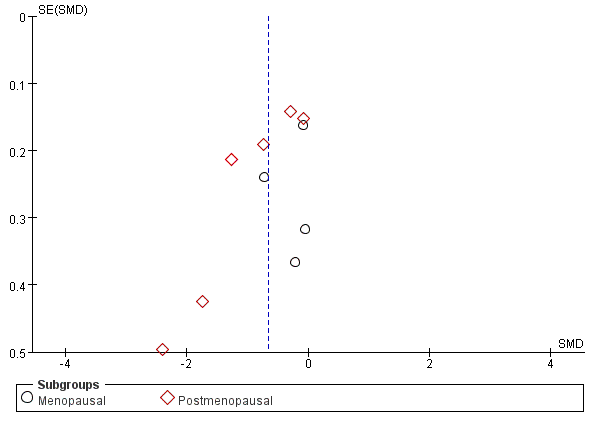
**

**Supplementary Table S4. Funnel plot for subgroup analysis by different menopausal state evaluating anxiety symptom.**


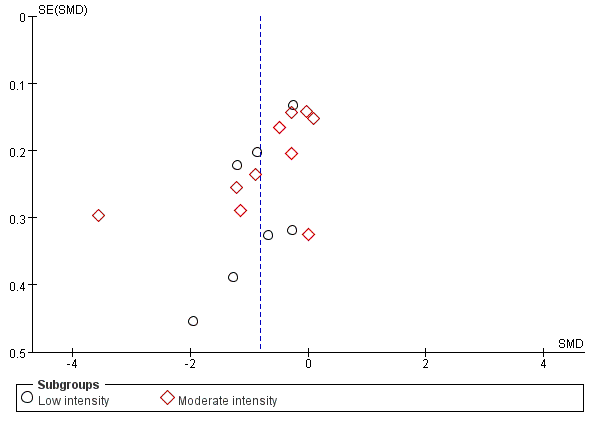


**Supplementary Table S5. Funnel plot for subgroup analysis by exercise intensity evaluating** **depression symptom.**

**
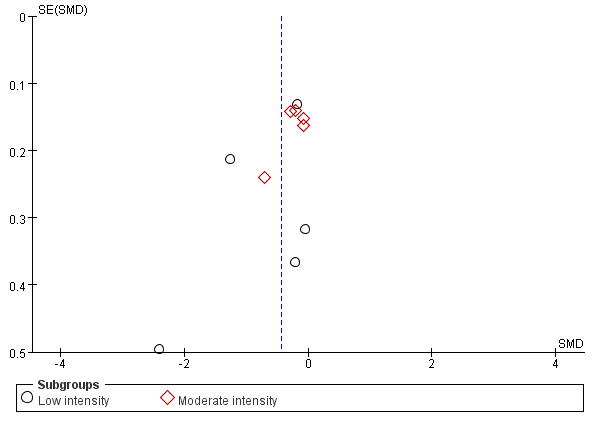
**

**Supplementary Table S6. Funnel plot for subgroup analysis by exercise intensity evaluating anxiety symptom.**


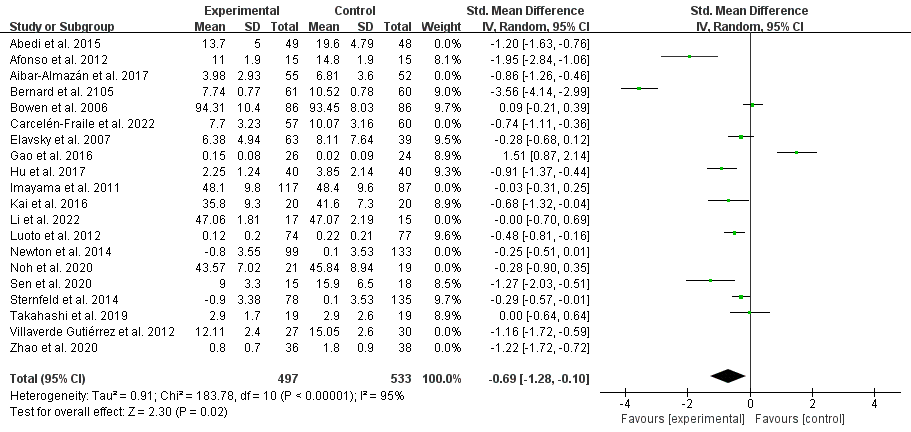


**Supplementary Table S7.** **Forest plot for sensitivity analyses evaluating depression symptom after removing studies with included women over 65 years old**


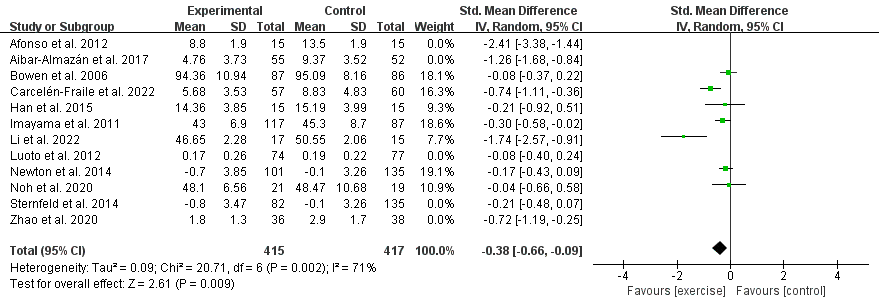


**Supplementary Table S8. Forest plot for sensitivity analyses evaluating anxiety symptom after removing studies with included women over 65 years old**


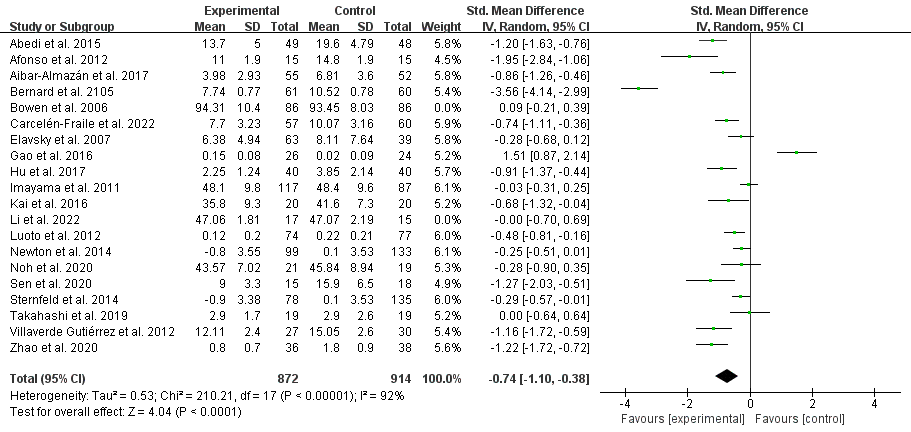


**Supplementary Table S9. Forest plot for sensitivity analyses evaluating depression symptom after removing trials that included women of the control group receiving non-exercise intervention**

**
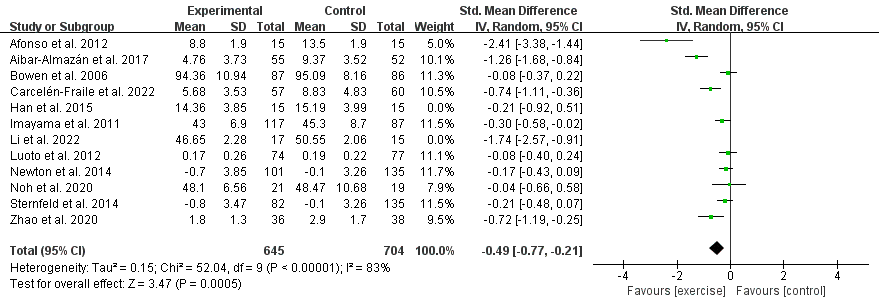
**

**Supplementary Table S10. Forest plot for sensitivity analyses evaluating anxiety symptom after removing trials that included women of the control group receiving non-exercise intervention**

**
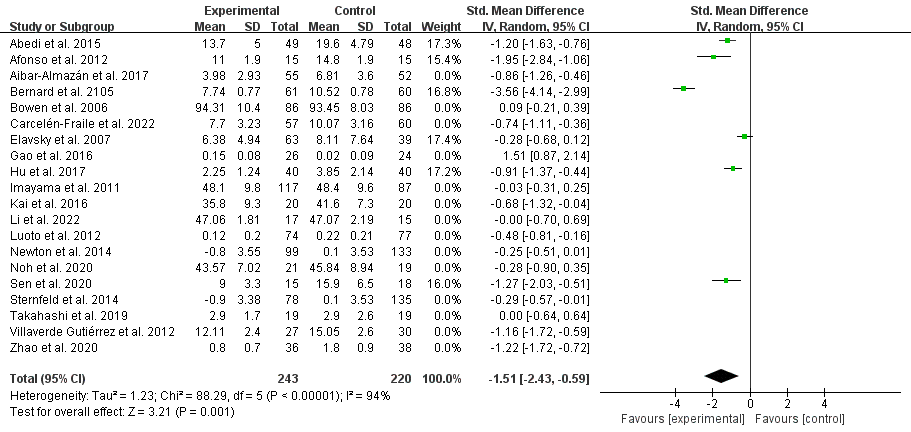
**

**Supplementary Table S11. Forest plot for sensitivity analyses evaluating depression symptom after including trials only using the BDI**
